# Iron metabolism and lymphocyte characterisation during Covid-19 infection in ICU patients: an observational cohort study

**DOI:** 10.1186/s13017-020-00323-2

**Published:** 2020-06-30

**Authors:** Giuliano Bolondi, Emanuele Russo, Emiliano Gamberini, Alessandro Circelli, Manlio Cosimo Claudio Meca, Etrusca Brogi, Lorenzo Viola, Luca Bissoni, Venerino Poletti, Vanni Agnoletti

**Affiliations:** 1 Anesthesia and Intensive Care Unit, AUSL Romagna – Bufalini Hospital, viale Ghirotti 286, 47521 Cesena, FC Italy; 2 Department of Respiratory Diseases & Allergy, Aarhus University Hospital, 8200 Aarhus, Denmark; 3 Department of Respiratory Diseases, AUSL Romagna - Morgagni Hospital, Forlì, Italy

**Keywords:** MeSH repository (3-10), Iron, COVID-19, SARS-CoV-2, Coronavirus, Critical care, Lymphocytes, Lymphopenia, Ferritins, Immunity, Coagulation

## Abstract

**Background:**

Iron metabolism and immune response to SARS-CoV-2 have not been described yet in intensive care patients, although they are likely involved in Covid-19 pathogenesis.

**Methods:**

We performed an observational study during the peak of pandemic in our intensive care unit, dosing D-dimer, C-reactive protein, troponin T, lactate dehydrogenase, ferritin, serum iron, transferrin, transferrin saturation, transferrin soluble receptor, lymphocyte count and NK, CD3, CD4, CD8 and B subgroups of 31 patients during the first 2 weeks of their ICU stay. Correlation with mortality and severity at the time of admission was tested with the Spearman coefficient and Mann–Whitney test. Trends over time were tested with the Kruskal–Wallis analysis.

**Results:**

Lymphopenia is severe and constant, with a nadir on day 2 of ICU stay (median 0.555 10^9^/L; interquartile range (IQR) 0.450 10^9^/L); all lymphocytic subgroups are dramatically reduced in critically ill patients, while CD4/CD8 ratio remains normal. Neither ferritin nor lymphocyte count follows significant trends in ICU patients. Transferrin saturation is extremely reduced at ICU admission (median 9%; IQR 7%), then significantly increases at days 3 to 6 (median 33%, IQR 26.5%, *p* value 0.026). The same trend is observed with serum iron levels (median 25.5 μg/L, IQR 69 μg/L at admission; median 73 μg/L, IQR 56 μg/L on days 3 to 6) without reaching statistical significance. Hyperferritinemia is constant during intensive care stay: however, its dosage might be helpful in individuating patients developing haemophagocytic lymphohistiocytosis. D-dimer is elevated and progressively increases from admission (median 1319 μg/L; IQR 1285 μg/L) to days 3 to 6 (median 6820 μg/L; IQR 6619 μg/L), despite not reaching significant results. We describe trends of all the abovementioned parameters during ICU stay.

**Conclusions:**

The description of iron metabolism and lymphocyte count in Covid-19 patients admitted to the intensive care unit provided with this paper might allow a wider understanding of SARS-CoV-2 pathophysiology.

## Background

Covid-19 has been declared a pandemic by World Health Organization (WHO) on the 11th of March. The high contagiousness and the previously unknown clinical features of this new viral infection put under pressure healthcare systems and clinicians worldwide.

Early reports from the Chinese province of Hubei described some predictive biomarkers for the clinical outcome of hospitalised patients, namely lymphopenia and the elevation of D-dimer, ferritin, interleukin 6 (IL-6), troponin and myoglobin, C-reactive protein (CRP) and lactate dehydrogenase (LDH) [1, 2]. LDH is a marker of parenchymal lung damage, and troponin and myoglobin are markers of myocardial and muscular involvement, while the remaining molecules belong to the group of positive acute-phase proteins (APP).

Ferritin is a crucial component of iron metabolism, one of the most ancestral systems of host protection from pathogen infections [3]. Iron is a micronutrient necessary for both energy production at a mitochondrial level and nucleic acid replication at cytoplasmic and nuclear level. For its scarcity in the human body and the fundamental processes in which it is involved, pathogens (bacterial, viral or fungal) compete with the host for iron availability in order to guarantee their own replication. When innate immunity is activated and cytokine cascades start, IL-1 and IL-6 stimulate hepcidin expression in the liver, reducing iron bioavailability by decreasing its gut absorption and hiding it into ferritin, a shell-like molecule deposited in macrophages. These mechanisms have been extensively reviewed in the literature [4–8].

Lymphopenia and specific T cell lineage affection are characteristic features of Covid-19 and have been correlated with poorer prognosis [9–11]. In previous coronavirus outbreaks, such as SARS, the peak of viral load occurred 7 days after symptoms development, followed by elevation in IL-6 and IL-8, nadir lymphocyte count and successive pulmonary infiltrates. This description suggests that clinical symptoms might be mediated by the immune system deregulation rather than direct viral damage [12]. The distribution of different subtypes of T cells in peripheral blood of symptomatic critical and non-critical Covid-19 patients has been described [13–16].

With this observational study, we aim to provide a detailed description of iron metabolism and lymphocyte subpopulations in ICU Covid-19 population. It can lead to a better understanding of the underlying physiopathology of this unknown disease, foreseeing some possible alternative therapeutic targets.

## Methods

### Study population

This is a single-centre retrospective observational cohort study. The first COVID-19-positive patient in our ICU has been admitted on the 5th of March 2020. Data collection goes from the 6th of March to the 6th of April 2020, following the local peak of epidemic. A protocol was established to test predictive biomarkers. On the day of ICU admission and then twice-a-week (on Monday and Thursday), every ICU patient was tested for ferritin, serum iron, transferrin and transferrin saturation (TfSat), soluble receptor of transferrin, CRP, D-dimer, LDH, troponin, lymphocyte count and characterisation of T cells (CD3, CD4 and CD8), B cells and NK cells. Then, data were divided in the following sub-categories: TI1-2 (first dosage made on ICU admission), TI3-6 (dosage between days 3 and 6 of ICU stay), TI 7-10 and TI 11-14. We observed their trends during the first 2 weeks of ICU stay. Due to laboratory restrictions, just 9 patients were tested for interleukin-6 (IL6) only on the day of admission [17].

### Inclusion criteria

Every SARS-CoV-2-positive patient (oropharyngeal swab or bronchoalveolar lavage sample, PCR test) admitted to our ICU was automatically enrolled in the study. Overall, 31 patients entered in the final data analysis fulfilling inclusion criteria. Importantly, we count ICU length of stay (LOS) from the day of admission in the first ICU: this means that dosages of patients transferred to our unit from other ICUs do not start from day 1.

### Data collection ended with the following criteria

Data collection was stopped after day 18 of ICU stay; iron-related data analysis was stopped or not taken into account for the final analysis if bacterial infection occurred, since we considered it a known confounding factor for inflammatory response; patients were discharged from our analysis on the date of extracorporeal membrane oxygenation (ECMO) start (3 patients) or death.

### Exclusion criteria

All patients admitted for strict clinical monitoring and discharged within 48 h from ICU; all admitted patients who tested negative for SARS-CoV-2; and no underage patients were included. Overall, 6 patients were initially tested but then excluded from the final analysis for the abovementioned criteria.

### Outcomes

The primary outcome was a descriptive analysis of iron metabolism values and lymphocyte count in ICU patients. Then, as a secondary outcome, we tried to correlate iron metabolism parameters and lymphocyte count with mortality or severity at the moment of ICU admission. Severity at the moment of ICU admission was quantified with two parameters: mean PaO_2_/FiO_2_ ratio [18] during the first 24 h (PFmed) and platelet-to-lymphocyte ratio [19] (PLR) on the day of ICU admission (data reported on Table 1).

**Table 1:** Demographic and general sample description

Reported cases	Male (%)	Female (%)
Gender (M/F)	25 (81)	6 (19)
Variables	Male (median (IQR))	Female (median (IQR))
Age (year)	62 (57–67)	71 (62–74)
BMI (kg/m^2^)	27.8 (25.3–31.1)	29.4 (29.3–30.8)
Symptoms-to-ICU (days)	9 (8–12)	7 (7–7)
In-hospital pre-ICU LOS (days)	2 (1–5)	2.5 (2–3)
ICU LOS	14 (9–19)	12.5 (11.2–16.7)
PFmed	195 (159.5–220)	165.5 (136.1–183.3)
PLR	436 (218–623)	305 (180–565)
CT scan (L:H)	6:15	2:2
Intubated patients (%)	22 of 25 (88)	6 of 6 (100)
Tracheostomy (%)	21 of 25 (84)	4 of 6 (67)
VAPs (%)	13 of 25 (52)	2 of 6 (33)
Pro-coagulative disorders (%)	3 of 25 (12)	0 of 6 (0)
Pnx (%)	7 of 25 (28)	1 of 6 (17)
CRRT (%)	10 of 25 (40)	1 of 6 (17)
Admitted from other centres (%)	12 of 25 (48)	3 of 6 (50)
Mortality (%)	7 of 21 (33) 4 men still admitted in our ICU	1 of 6 (17)

*BMI* body mass index, *CRRT* continuous renal-replacement therapy, *CT scan* computed tomography (only those with both images and radiologic report available—H stays for high-elastance pattern and L stays for low-elastance pattern), *ICU* intensive care unit, *IQR* interquartile range, *LOS* length of stay, *Mortality* 21 men over 25 enrolled because 4 are still in our ICU, *PFmed* means PaO_2_/FiO_2_ ratio during the first 24 h of ICU stay, *PLR* platelet-to-lymphocyte ratio on the day of ICU admission, *Pnx* pneumothorax, *Pro-coagulative disorders* deep-vein thrombosis, ischaemic stroke or massive pulmonary embolism, *VAP* ventilator-acquired pneumonia

### Measurement technology

Iron parameters were tested with Cobas analyser systems (RocheⓇ), while lymphocyte subpopulations with the Navios EX flow cytometer (Beckman CoultrerⓇ).

### Statistical analysis

The formula used to estimate TfSat (TfSat = serum iron/(transferrin × 1.42) × 100) is not reliable for ferritin values above 1200 μg/L: in this case, those values were not included in final analysis. Statistical analysis was performed using the software IBM SPSS 22.0. Data are reported as mean with standard deviation (std. dev.), median with interquartile range (IQR), number and percentage, depending on underlying distribution. Student’s *t* test, Mann–Whitney, Kruskal–Wallis, Spearman correlation and *χ*^2^ tests were used for statistical analysis.

## Results

Table 1 summarises anthropometric features and characteristics of our sample. The intubation rate is particularly elevated because in our Hospital are present sub-intensive care units and because ICU stay lower than 48 h (only for strict monitoring purposes) was an exclusion criteria. It is therefore likely the analysis accounts for a population of severely affected patients. CT scan patterns of low (L) or high (H) elastance refer to the phenotypes proposed by Gattinoni et al. [20]. Due to the exiguity of our sample and the different focus of our work, we intentionally did not test *p* values for the clinical features reported in Table 1.

**Fig. 1:**
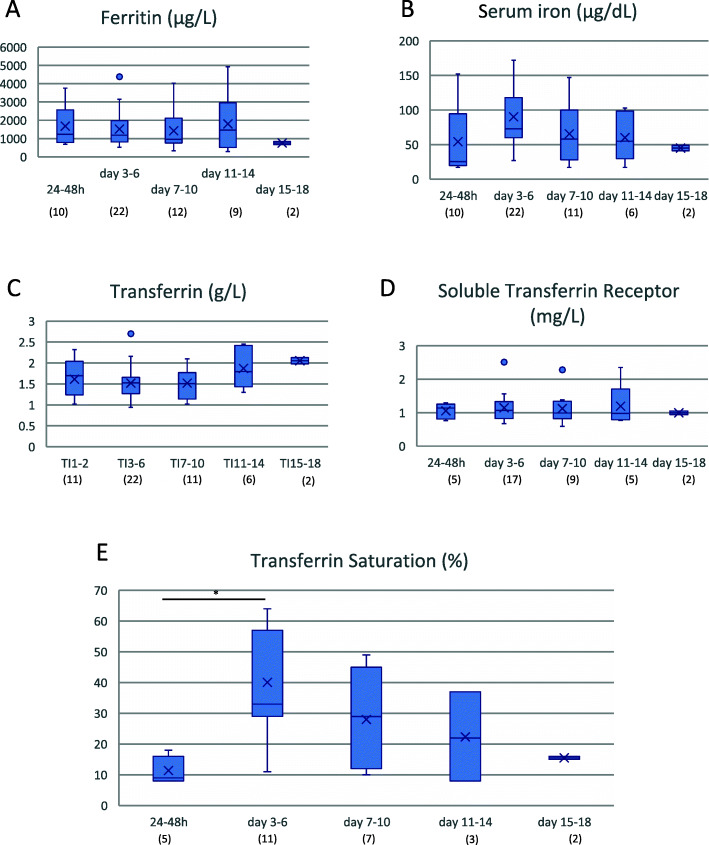
Iron parameters. Distribution over time of the tested iron parameters with their units of measure. Box plots indicate median with interquartile range, and dots indicate outliers. Groups specify the timing by which the dosage was effectuated form the moment of ICU admission, and round brackets in the lower line indicate the number of patients tested for each group. In **e**, asterisk indicates significant difference (*p* value > 0.05)

A significant increase of TfSat levels (%) occurs between the first 48 h of ICU stay and days 3 to 6 (Mann–Whitney, *p* value = 0.026, Fig. 1). No other significant trends and modifications of iron parameters and lymphocyte subpopulations were found during ICU stay. Lymphocytes and their subpopulations do not show significant trends of modification either.

**Fig. 2:**
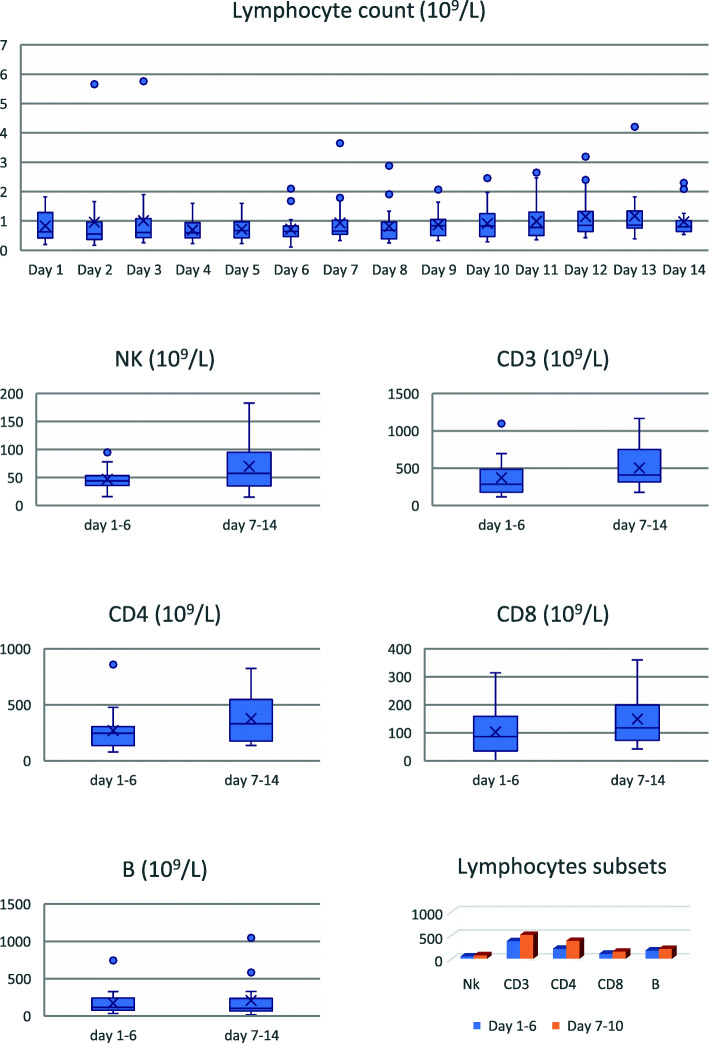
Lymphocytes count. Distribution over time of lymphocytes and their tested subgroups, with their units of measure. Box plots indicate median with interquartile range, and dots indicate outliers. Groups specify the timing by which the dosage was effectuated form the moment of ICU admission. The number of patients tested is reported in Tables 3 and 4. Lymphocyte subsets merge all the subgroups to make their trend over time more readable

Trends and modifications of iron parameters are reported in Fig. 1, while trends and modifications of lymphocytes count and lymphocytic subgroups are reported in Fig. 2.

Looking to secondary outcomes, neither iron parameters nor lymphocyte count correlate with mortality, PFmed or PLR. Their values are reported in Tables 2, 3 and 4.

**Table 2:** Iron parameters over time

	Days 1–2	Days 3–6	Days 7–10	Days 11–14	Days 15–18
Ferritin (μg/L)	1236.5 (1648) 10	1185 (1018) 22	964.5 (1251) 12	1470 (1866) 9	755 (172) 2
Serum iron (μg/L)	25.5 (69) 10	73 (56) 22	58 (61) 11	55 (63) 6	45 (8) 2
Transferrin (g/L)	1.67 (0.73) 11	1.82 (0.38) 22	1.56 (0.58) 11	1.88 (0.93) 6	2.06 (0.15) 2
Transferrin Saturation (%)	9 (7) 5	33 (26.5) 11	29 (26.5) 7	22 (22) 3	16 (1) 2
Transferrin Receptor (mg/L)	1.14 (0.40) 5	1.07 (0.49) 17	0.99 (0.47) 9	0.98 (0.58) 5	1.00 (0.11) 2

Data are all expressed as median, interquartile range between brackets and number of measured cases in the second line

**Table 3:** Lymphocyte subgroups over time

	Days 1–7	Days 8–14
NK (10^9^/L)	44 (14.25) 17	57.5 (50.5) 16
T—CD3 (10^9^/L)	286 (284.75) 17	409.5 (422.5) 16
T—CD4 (10^9^/L)	246 (161) 16	331 (364) 17
T—CD8 (10^9^/L)	86.5 (121) 18	117.5 (117) 16
B	116 (146) 15	102 (161) 17

Data are all expressed as median, interquartile range between brackets and number of measured cases in the second line

**Table 4:** Total lymphocyte count over time

	D1	D2	D3	D4	D5	D6	D7	D8	D9	D10	D11	D12	D13	D14
Lympho (10^9^/L)	0.630 (0.780) 14	0.555 (0.450) 18	0.610 (0.605) 20	0.600 (0.493) 21	0.670 (0.540) 22	0.645 (0.330) 22	0.655 (0.480) 22	0.680 (0.550) 22	0.840 (0.493) 19	0.830 (0.750) 21	0.780 (0.715) 21	0.850 (0.650) 18	0.860 (0.510) 17	0.800 (0.340) 15

Data are all expressed as median, interquartile range between brackets and number of measured cases in the second line

The already described survival predictors in general hospital population (LDH, troponin, CRP and D-dimer) are not significantly associated with outcome, PLR or PFmed in our ICU population and do not show significant trends. Data are reported in Table 5.

**Table 5:** Clinical biomarkers over time

	Days 1–2	Days 3–6	Days 7–10	Days 11–14	Days 15–18
D-dimer (μg/L)	1319 (1285) 11	6820 (6619) 19	3718 (4631) 14	2959 (2923) 12	2391 (2693) 10
LDH (U/L)	476 (226) 13	451 (205) 26	425 (218) 22	404 (134) 19	376 (223) 9
CRP (mg/L)	116 (46) 13	192 (72) 21	52 (190) 16	129 (129) 17	111 (117) 10
Troponin T (ng/L)	19 (15) 7	21 (10) 19	18 (10) 15	15 (18) 13	18 (12) 7
IL6 (pg/mL)	42.5 (47.4) 9				

**Fig. 3:**
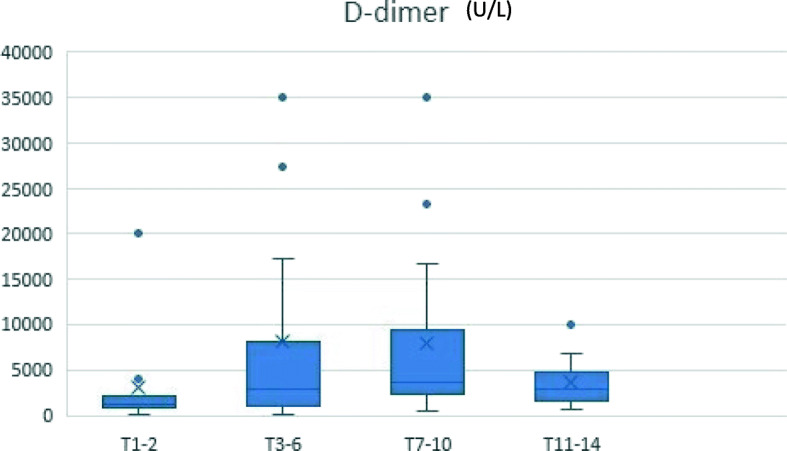
D-dimer levels. D-dimer levels over time from the moment of ICU admission. It is observable a non-significant trend towards increase after the first 48 h. It might be relevant to explain the increased tendency to hypercoagulability of Covid-19 patients. Box plots indicate median with interquartile range, and dots indicate outliers. Groups specify the timing by which the dosage was effectuated form the moment of ICU admission

D-dimer shows a non-significant tendency to increase after ICU admission (Kruskal–Wallis, *p* value = 0.108, Fig. 3). IL6 elevation is lower than reported by other studies on critically ill Covid-19 patients [21]. We found a debile correlation between IL6 levels and lymphocyte count on the day of ICU admission (8 cases, Spearman rho 0.714, *p* value = 0.047), but the analysis is limited by the low amount of cases.

## Discussion

Cytokines release hyper-expresses hepcidin, leading to ferroportin internalisation and reduced iron absorption and availability in body fluids [22, 23]. Serum iron and TfSat are known to decrease early after infections, inhibiting iron availability to the pathogen, but then return to almost-normal values within 7–10 days [24, 25]. The role of dysregulated iron response in Covid-19 has been recently reviewed [26].

Serum iron does not show significant modifications, but it follows the same trend of TfSat being part of the formula to calculate it. They both remain lower than the normal reference values during the whole infection. It is difficult to hypothesise the reason of their trend without proper laboratory research: the dramatic low levels at ICU admission might be expected, but the following increase during an acute phase of inflammatory response seems counter-intuitive. The described patients arrived in our ICU after a median of 7 to 9 days from symptoms onset: the physiologic progressive restoration of normal TfSat after 1 week of infection and the dysregulated cytokine response might contribute to TfSat elevation. Also, it has been described that acute respiratory distress syndrome (ARDS) causes reduced serum iron levels [27]: intubated Covid-19 patients suffer of this syndrome, while protective ventilation might theoretically restore normal iron levels.

Ferritin is a very early and non-specific indicator of inflammation. It resulted to be the first severely elevated biomarker together with lymphopenia [1]: thus, its early dosage in at-home symptomatic patients might be extremely useful in individuating those who can benefit of early hospital admission. After its initial rise, ferritin can take longer than a month to normalise after an infection [24]. Thus, it remains elevated during ICU stay of Covid-19 patients. Despite being apparently futile, its dosage constitutes the key element to suspect secondary haemophagocytic lymphohistiocytosis (sHLH). sHLH is a frequently misdiagnosed syndrome related to viral infections and thus of primary importance in this Covid-19 pandemic. Overall, despite unable of a more detailed description, we demonstrate how iron metabolism is deranged in severe forms of Covid-19.

Lymphocytes are constantly reduced in ICU Covid-19 patients with respect to reference values. All the subsets are also dramatically reduced, more than reported by other recent publications referring to non-ICU populations [9, 28]. We observe a conserved CD4/CD8 ratio. The nadir of lymphopenia is on day 2 of ICU stay; a progressive tendency towards normalisation is more evident in patients experiencing positive outcome and ICU discharge. Similar timing of lymphocyte modifications was observed during severe acute respiratory disease (SARS) outbreak, despite with less dramatic reduction [29, 30]. Being referred to critically ill patients, this more severe reduction of all lymphocyte subgroups might be an indicator of severity. To date, no other publications are available about ICU populations on lymphocytes subgroups.

Being such a specific subset of critically ill patients, many significant differences were not observable between survivors and non. These data might be relevant to researchers for a better understanding of the altered immunologic response in severely affected patients and to ICU clinicians.

The study is affected by some limitations. The sanitary emergency and the previously unknown characteristics of this disease led to many difficulties. During the initial phase, it has been difficult to create an effective protocol shared between the operators and some data have been missed. We have frequently acted as a backup hospital to relieve other overloaded ICUs nearby: this means that, for a part of the reported cases, the first few days of ICU stay occurred in a different unit and the dosage of relevant markers was missed during that initial phase. Being a monocentric study conceived during an emergency phase, the sample size is limited and cannot be increased: this led to the impossibility to detect and describe some more subtle physiologic processes. Finally, slightly different therapeutic approaches have been applied to patients following ongoing findings and different complications (ventilator-acquired pneumonia, pro-coagulative states, renal and hepatic insufficiency) have affected patients’ evolution: it is not possible to quantify how these differences affected iron metabolism and lymphocyte count.

## Conclusions

We describe iron metabolism, lymphocyte subgroups count and other biomarkers in critically ill Covid-19 patients. This might be relevant for clinicians dealing with critical patients and provide further hints about the pathophysiology of this disease.

Iron metabolism has been repeatedly proposed as a potential therapeutic target during infections [31, 32]. Pharmacologic advances have made available safer iron chelators [33]. This testifies the relevance of the topics investigated and how they might contribute to the development of novel therapeutic strategies against Covid-19.

## Data Availability

The datasets used and analysed during the current study are available from the corresponding author on reasonable request.

## Abbreviations

APP: Acute phase-proteins
ARDS: Acute respiratory distress syndrome
CRP: C-reactive protein
CRRT: Continuous renal replacement therapy
ECMO: Extracorporeal membrane oxygenation
HLH: Haemophagocytic lymphohistiocytosis
IL-6: Interleukin 6
IQR: Interquartile range
LDH: Lactate dehydrogenase
LOS: Length of stay
MOF: Multiple organ failure
PCR: Polymerase chain reaction
PFmed: Mean PaO_2_/FiO_2_ ratio during the first 24 h of ICU admission
PLR: Platelet-to-lymphocyte ratio on the day of ICU admission
PPE: Personal protective equipment
SARS: Severe acute respiratory syndrome
SARS-CoV-2: SARS coronavirus type 2
TfSat: Transferrin saturation (%)
VAP: Ventilator-acquired pneumonia
WHO: World Health Organization
